# The randomised uterine septum transsection trial (TRUST): design and protocol

**DOI:** 10.1186/s12905-018-0637-6

**Published:** 2018-10-05

**Authors:** J. F. W. Rikken, C. R. Kowalik, M. H. Emanuel, M. Y. Bongers, T. Spinder, J. H. de Kruif, K. W. M. Bloemenkamp, F. W. Jansen, S. Veersema, A. G. M. G. J. Mulders, A. L. Thurkow, K. Hald, A. Mohazzab, Y. Khalaf, T. J. Clark, M. Farrugia, H. A. van Vliet, M. S. Stephenson, F. van der Veen, M. van Wely, B. W. J. Mol, M. Goddijn

**Affiliations:** 1 Center for Reproductive Medicine, Academic Medical Centre, University of Amsterdam, PO Box 22700, 1100 DE Amsterdam, The Netherlands; 2 University Medical Center Utrecht, Heidelberglaan 100, 3584 Utrecht, The Netherlands; 3 Maxima Medical Centre, de Run 4600, 5504 DB Veldhoven, The Netherlands; 4 Leeuwarden Medical Centre, Henri Dunantweg 2, 8934 AD Leeuwarden, the Netherlands; 5 Canisius Wilhelmina Hospital, PO Box 9015, 6500 GS Nijmegen, The Netherlands; 6 University Medical Centre Leiden, Albinusdreef 2, 2333 ZA Leiden, The Netherlands; 7 Erasmus Medical Centre, ‘s-Gravendijkwal 230, 3015 CE Rotterdam, The Netherlands; 8 Oslo University Hospital, P. O. Box 4950, Nydalen, N-0424 Oslo, Norway; 9 Avicenna research institute Teheran, PO Box: 19615-1177, Teheran, Postal code: 1936773493 Iran; 10 Guy’s hospital, Great maze pond, London, SE1 9RT UK; 11 Birmingham women’s hospital, Mindelsohn Way, Birmingham, West Midlands B15 2TG UK; 12 East Kent Hospitals University, Ethelbert road, Canterbury, Kent, CT1 3NG UK; 13 Catharina hospital, Michelangelolaan 2, 5623 EJ Eindhoven, the Netherlands; 14 University of Illinois Hospital, 1740 W Taylor St, Chicago, IL 60612 USA; 15 The Robinson Institute, School of Paediatrics and Reproductive Health, University of Adelaide, Adelaide, Australia

**Keywords:** Septate uterus, Recurrent miscarriage, Subfertility, Hysteroscopic septum resection, Randomised controlled trial

## Abstract

**Background:**

A septate uterus is a uterine anomaly that may affect reproductive outcome, and is associated with an increased risk for miscarriage, subfertility and preterm birth. Resection of the septum is subject of debate. There is no convincing evidence concerning its effectiveness and safety. This study aims to assess whether hysteroscopic septum resection improves reproductive outcome in women with a septate uterus.

**Methods/design:**

A multi-centre randomised controlled trial comparing hysteroscopic septum resection and expectant management in women with recurrent miscarriage or subfertility and diagnosed with a septate uterus. The primary outcome is live birth, defined as the birth of a living foetus beyond 24 weeks of gestational age. Secondary outcomes are ongoing pregnancy, clinical pregnancy, miscarriage and complications following hysteroscopic septum resection. The analysis will be performed according to the intention to treat principle. Kaplan-Meier curves will be constructed, estimating the cumulative probability of conception leading to live birth rate over time. Based on retrospective studies, we anticipate an improvement of the live birth rate from 35% without surgery to 70% with surgery. To demonstrate this difference, 68 women need to be randomised.

**Discussion:**

Hysteroscopic septum resection is worldwide considered as a standard procedure in women with a septate uterus. Solid evidence for this recommendation is lacking and data from randomised trials is urgently needed.

**Trial registration:**

Dutch trial registry (NTR1676, 18th of February 2009).

**Electronic supplementary material:**

The online version of this article (10.1186/s12905-018-0637-6) contains supplementary material, which is available to authorized users.

## Background

A septate uterus is a congenital uterine anomaly where the uterus is divided into two cavities. A septate uterus is associated with reduced fertility (RR 0.86; 95% CI 0.77–0.96), increased miscarriage rates (RR 2.9; 95% CI 2.0–4.1) and preterm delivery (RR 2.1; 95% CI 1.5–3.1) [1].

The mechanisms behind the negative effect of the septum on fertility and pregnancy outcome have not yet been clarified. Suggestions are that the septum is a poor site for embryonic implantation due to the assumed poor vascularization, decreased sensitivity to preovulatory changes of the endometrium overlying the septum, uncoordinated contractility of the septum, or a local defect of vascular endothelial growth factor (VEGF) receptors in the endometrium covering the septal area [2–5]. A septate uterus is usually ascertained through recurrent pregnancy loss or subfertility, and occasionally by other complaints such as dysmenorrhoea or preterm birth. Approximately 6% of women with recurrent miscarriage and 3.5–6.4% of subfertile women, has a septate uterus [6–8]. .In comparison, this prevalence is 2–3% in the general female population [6, 7].

Although evidence for the effectiveness of resecting the uterine septum is limited, resection is considered standard care in many countries. The evidence is mainly based upon retrospective studies, in which improved pregnancy chances and live birth rates after hysteroscopic septum resection have been suggested [9–17].

The major flaw in most of the abovementioned studies is obviously the before/after design, since this will always favour the tested intervention as the prognosis without the intervention is usually good [18, 19]. At this moment, in view of the limited quality of the performed studies it thus remains unclear whether removal of the septum will eliminate negative effects –if any- of a septate uterus, and whether the procedure does not cause harmful side effects [20] .

To assess whether surgical intervention in women with (recurrent) miscarriage, subfertility or preterm birth and a septate uterus will improve reproductive outcome, we propose The Randomised Uterine Septum Trial (TRUST).

## Methods

### Design

The Randomised Uterine Septum Trial (TRUST) is a multicentre randomised controlled trial in women with a septate uterus (NTR 1676). Women are randomised to hysteroscopic septum resection or expectant management. Recruitment has started since October 2009 and is ongoing.

### Trial population

**Fig. 1:**
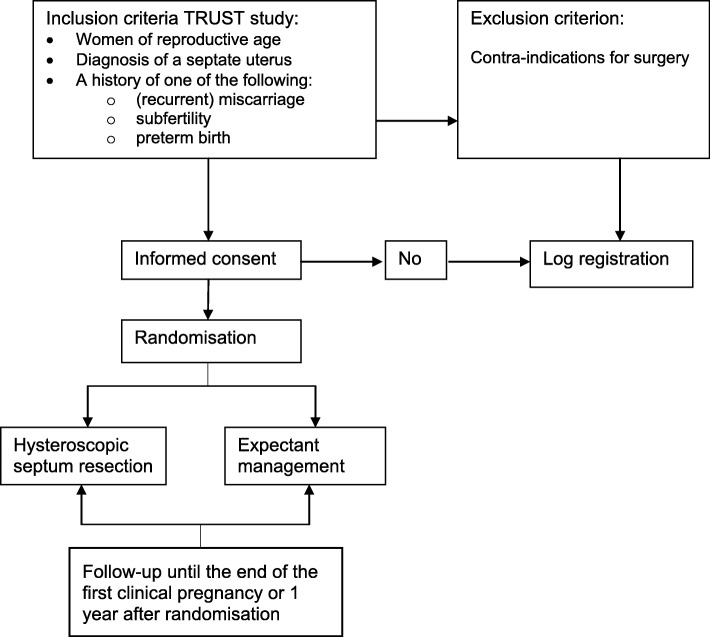
Flowchart

The study population consists of women of reproductive age with a septate uterus and a history of (recurrent) miscarriage, subfertility or preterm birth (Fig. 1). Women with a contraindication to surgery are excluded for the trial. When the study started in 2008, the study population consisted of women with recurrent miscarriage and a septate uterus. During the course of the trial we found out that it was difficult to recruit and randomize women for the study. Since essentially the population is women with a septate uterus and a wish to conceive, we decided to broaden the inclusion criteria to make an effort to identify these women more easily. Thus in 2011 we extended the inclusion criteria into women with a septate uterus and recurrent miscarriage and/or subfertility, and in 2015 the inclusion criteria were extended to women with (recurrent) miscarriage, subfertility or preterm birth. All amendments were approved by the ethical committee of the Academic Medical Centre (AMC), Amsterdam. Recurrent miscarriage is defined as two or more, not necessarily consecutive, pregnancy losses before 20 weeks of gestational age. Subfertility is defined as the inability to conceive for a minimal period of one year of trying to conceive. The definition of preterm birth is birth before a gestational age of 37 complete weeks. Only women with an active wish to conceive are eligible for the trial. Following the most recent ESHRE-ESGE classification, a septate uterus is defined as all cases with an abnormal resorption of the midline septum, a normal outline of the uterus and an internal indentation at the fundal midline exceeding 50% of the uterine wall thickness regardless of the size of the septum [21]. Over the years there has been a lot of discussion about the classification system of uterine anomalies and ideas about the best definition of the septate uterus have changed [22]. After the following publication of the ESHRE-ESGE classification, we have adjusted the definition in the study protocol accordingly to these new insights, but it did not change our patient population [21].The presence of a uterine septum is ascertained by HSG, 3D ultrasound (3D-US), MRI, saline or gel infusion sonohysterography or hysteroscopy combined with laparoscopy [23–26].

### Recruitment procedure

Women are recruited from outpatient clinics located in the Netherlands, Iran and the United States of America. Women are invited to take part in the study when a septate uterus is diagnosed and when they meet the inclusion criteria.

### Ethics and trial registration

Approval for this study and all subsequent amendments have been obtained from the Medical Ethical Committee of the Academic Medical Centre (IDS NL24082.018.08 MEC Academic Medical Centre, Amsterdam) The Netherlands. Local approval is obtained at all participating centres or will be obtained before the start of recruitment. The trial is registered within the Dutch trial registry (NTR1676).

Written informed consent is obtained from women fulfilling the inclusion criteria, prior to randomization.

### Randomization

Once eligibility for the trial has been confirmed and women have given informed consent they are randomised via a web-based central randomization system to receive either hysteroscopic septum resection or expectant management .

### Hysteroscopic septum resection

The intervention is hysteroscopic septum resection. The choice for an instrument and distension medium may vary per hospital and depends on the preference of the surgeon and instruments available. Analgesia during surgery can be either general anaesthesia or a loco-regional technique depending on the preference of the woman and whether concomitant surgery, in the form of laparoscopy, is scheduled. To prevent uterine perforation during surgery, a laparoscopy or ultrasound has to be performed simultaneously [27]. The choice for laparoscopy or ultrasound depends on the local hospital protocol. To assess the results of the resection, a diagnostic hysteroscopy is performed 6–8 weeks postoperatively in an outpatient setting. When a secondary surgery is needed, this will be specified.

### Expectant management

The control group will receive expectant management. Additional interventions, such as for example aspirin or heparin in case of co-existing antiphospholipid syndrome, or additional artificial reproductive techniques in women with subfertility, are allowed in both groups and will be registered. Should the first pregnancy after randomization result in a miscarriage, or should pregnancy not occur after one year of follow-up, women are free to opt for additional therapy, including hysteroscopic septum resection.

### Outcome measures

The primary outcome measure is live birth, defined as the birth of a living foetus beyond 24 weeks of gestational age.

Secondary outcomes are ongoing pregnancy, clinical pregnancy, miscarriage, pregnancy outcomes as placental abruption, uterine rupture, preterm birth and mode of delivery (vaginal versus caesarean section), perinatal morbidity, mortality and complications following hysteroscopic septum resection, such as uterine perforation, fluid overload and endometritis.

### Follow up

Follow up will take place for at least one year. Women who will conceive in that period will be followed for the course of that pregnancy. Woman allocated for expectant management, have the opportunity to opt for a hysteroscopic septum resection after one year of follow up, or when the next pregnancy results in a miscarriage. An additional timeline shows more detail on the timeline of study (see Additional file 1).

### Statistical analysis

All analyses will be performed according to an intention to treat basis. The primary outcome, live birth, will be compared between the intervention and control group.

Relative risks and 95% confidence intervals will be calculated for the relevant outcome measures. Time to conception, resulting in live birth will also be assessed by means of life table analysis. To estimate the cumulative probability of conception resulting in live birth rate over time Kaplan-Meier curves will be constructed. The risk of premature birth will be estimated stratified for gestational age. The relative risk for obstetrical and surgical complications will be calculated.

### Sample size

The sample size is based on retrospective studies, anticipating an improvement of the live birth rate from 35% without surgery to 70% with surgery. Using a two-sided test, an alpha-error of 5% and a beta-error of 20%, two groups of 31 women are needed to demonstrate this difference. Anticipating lost-to-follow up and protocol violation, an additional 10% is needed. Thus, 68 women need to be randomised.

## Discussion

Until this date, hysteroscopic septum resection of a septate uterus is standardly being performed worldwide in women of reproductive age and a wish to conceive [28, 29].

The evidence for this recommendation is based on retrospective and prospective comparative studies that suggest that restoration of the uterine morphology can potentially have a positive effect on live birth rate [9–17]. Most retrospective studies used their own participants as a control group. To our knowledge, over the years nine prospective comparative studies have been published. The studies describe miscarriage, pregnancy or live birth rate in women with a septate uterus who consented to hysteroscopic septum resection, compared with women who chose expectant management. These studies published contradictory findings, with some studies showing significant higher pregnancy rate in women with a septate uterus who were treated with surgery [12, 13, 30, 31], while other studies found no significant difference [32–35]. Thus, equipoise still exists and data from randomised trials are needed to provide definitive proof of effectiveness of a septum resection, or the lack thereof. In view of the major improvements in pregnancy rates reported by the non-comparative retrospective studies, we require a low number of participants to exclude a difference of 35% or more. By broadening our inclusion criteria we did not need to re-calculate the sample size since our primary population, i.e. women of reproductive age with a septate uterus, remained the same. Over the years, many studies have been published about the classification of uterine anomalies and the interrater disagreement in diagnosing the septate uterus still exists [36, 37]. We have changed the definition of the septate uterus according to new insights leading to the ESHRE ESGE classification, and in retrospect all women meet these new criteria [21].

To our knowledge the TRUST trial is the first registered multi centre randomised controlled trial designed to assess whether hysteroscopic septum resection improves live birth rate (NTR1676). If hysteroscopic septum resection proves to be without effect on reproductive outcome, this would imply a major change in treatment policy and adapted guidelines.

## Additional file

Additional file 1: Timeline of study. (PDF 12 kb)

## Abbreviations

3D US: 3D Ultrasonography
TRUST: The Randomised Uterine Septumtranssection Trial
VEGF: Vascular endothelial growth factor
