# Supplementary figures and images for: The randomised uterine septum transsection trial (TRUST): design and protocol
Source: BMC Womens Health. 2018 Oct 5;18:163. doi: 10.1186/s12905-018-0637-6 (PMC6173848; doi:10.1186/s12905-018-0637-6)

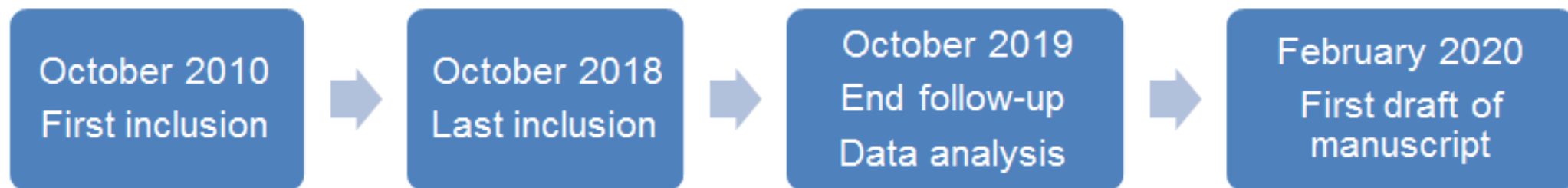

Supplement: Supplementary file 1 — Timeline of study. (PDF 12 kb) [file 12905_2018_637_MOESM1_ESM.pdf]
